# Sex differences in atrial fibrillation incidence among individuals with metabolic syndrome: a narrative mini review

**DOI:** 10.3389/fcvm.2026.1969128

**Published:** 2026-10-28

**Authors:** Stefano Bonapace, Giulio Molon, Paolo Tosi, Esther Campopiano, Alessandro Costa, Antonio Pepe, Riccardo Morandin, Alessio Marinelli, Giovanni Targher, Alessandro Mantovani

**Affiliations:** 1 Department of Cardiology, IRCCS Sacro Cuore - Don Calabria, Negrar di Valpolicella, Italy; 2 Department of Medicine, University of Verona, Verona, Italy; 3 Metabolic Diseases Research Unit, IRCCS Sacro Cuore - Don Calabria Hospital, Negrar di Valpolicella, Italy; 4 Department of Cardiology, AOOR Villa Sofia-Cervello, Palermo, Italy

**Keywords:** atrial fibrillation, estrogen, metabolic syndrome, sex difference, testosterone

## Abstract

**Background:**

Sex differences influence the development and clinical expression of many chronic cardiometabolic diseases. Atrial fibrillation (AF), the most common sustained arrhythmia, frequently coexists with metabolic syndrome (MetS), yet whether MetS confers different AF risk in men and women remains incompletely understood.

**Content:**

Epidemiological evidence consistently demonstrates that MetS increases the risk of incident AF by approximately 1.4- to 1.9-fold. Among MetS components, elevated blood pressure and abdominal obesity show the strongest associations with AF onset. Most studies also reveal that men have higher rates of AF than women across all age groups, although AF incidence in women rises sharply after menopause. In individuals with MetS, similar sex-related trends are observed: men typically exhibit a higher and earlier AF incidence, whereas women experience a delayed but steep increase in older age.

Biological differences—particularly sex hormone–driven variations in fat distribution, lipid metabolism, insulin sensitivity, and inflammation—may underlie these patterns. Premenopausal women tend to accumulate subcutaneous, metabolically benign fat, whereas men preferentially store visceral fat, promoting earlier metabolic dysfunction and AF susceptibility. After menopause, declining estrogen levels shift women toward central adiposity and cardiometabolic deterioration, narrowing the AF incidence gap. Sex-related differences in autonomic balance and adiposity-related sympathetic activation may further modulate AF risk.

**Conclusion:**

While MetS increases AF risk in both sexes, accumulating evidence suggests that the magnitude, timing, and underlying mechanisms of this risk differ between men and women. Better integration of sex-specific biological pathways into AF prevention strategies is warranted.

## Graphical Abstract

**Figure:**
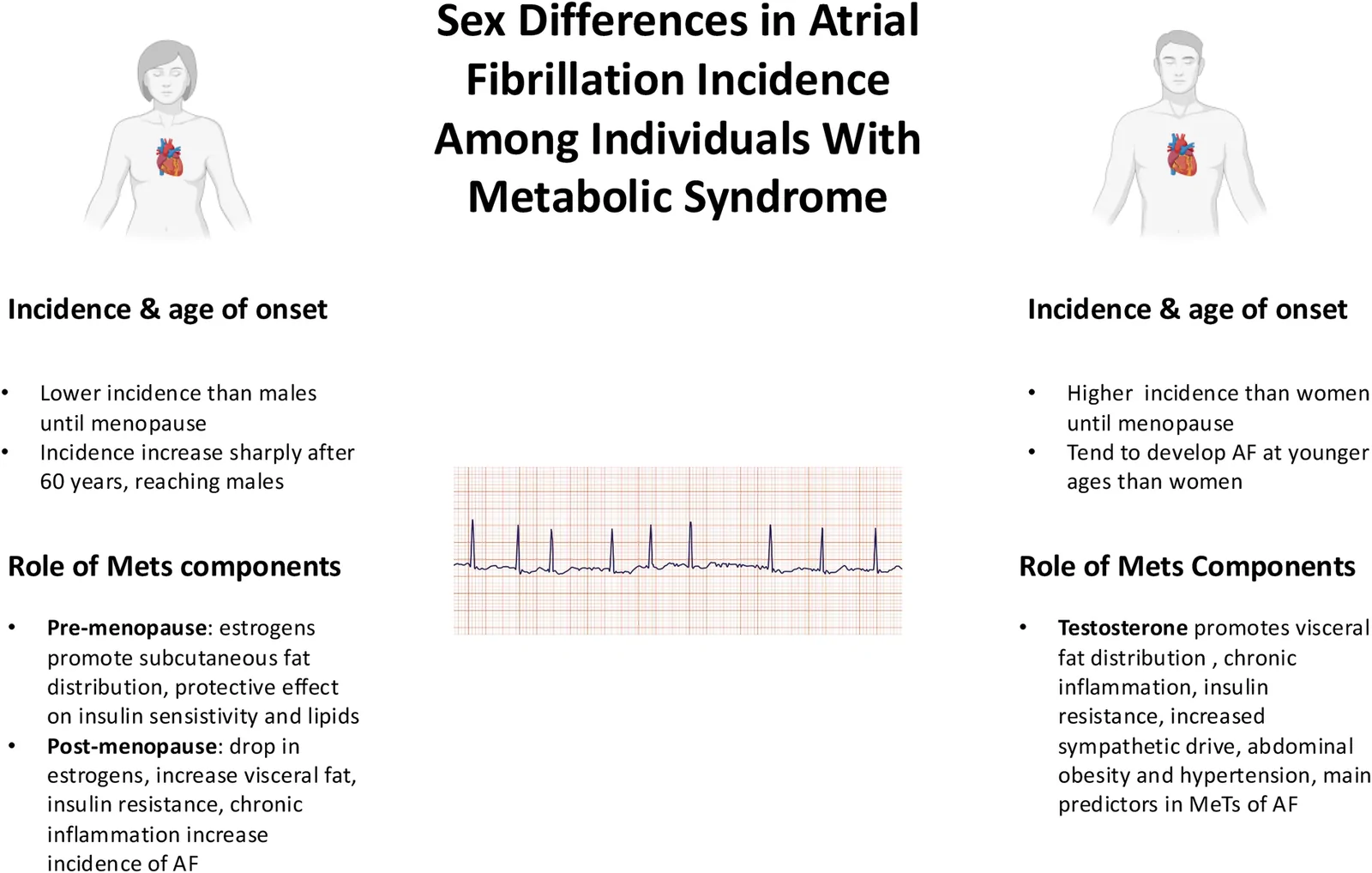

## Introduction

Recognizing sex and gender differences in chronic diseases is crucial to understanding the biological and sociocultural factors that can influence health outcomes (1, 2). The terms “sex” and “gender”, although often used interchangeably, refer to different concepts. The former indicates the biological differences between males and females, while the latter includes the social behaviours of being male or female (1, 2). Both have significant roles in determining the risk, presentation, and progression of various chronic diseases, as well as the responses to treatment (1, 2). Cardiovascular and cardiometabolic diseases are among the leading causes of death worldwide, with substantial differences between men and women in their development and outcomes (1, 2). In this narrative review, we specifically discuss the literature data on whether there are sex differences in the onset of atrial fibrillation (AF) in patients with metabolic syndrome (MetS), and discuss the putative underlying mechanisms. In contrast, gender differences are beyond the scope of this narrative review. However, differences in both physiological and psychosocial environmental factors (e.g., smoking history, alcohol consumption, food habits, and physical activity) between women and men may result in differential effects on the incidence of AF both in the general population and in individuals with MetS (3).

### Epidemiology of atrial fibrillation and MetS definition

AF is the most common sustained arrhythmia, which significantly increases the risk of dying or developing ischemic stroke and congestive heart failure, making it a public health concern worldwide (4). The global prevalence of AF in adults is between 2% and 4% (5), but an approximately two-fold increase in AF prevalence (6) is expected in subsequent years (7, 8), mainly due to increasing longevity of the general adult population and the improvement in AF diagnosis and treatment (7, 8). The prevalence of AF is usually greater in men than in women (4–8), and one in three European individuals will develop AF at an index age of 55 years (9). Men tend to develop AF at younger ages, and the overall lifetime risk of developing AF is higher in men than in women (4–9). The Framingham Heart Study has shown that, even after adjusting for known risk factors for AF, men have a higher AF incidence than women across all age groups, but in women, there is an increase in incident AF that parallels men in older ages (9). In a meta-analysis of 30 studies (involving about 4.4 million participants), of which five were randomized controlled trials and 24 were observational studies, women with AF had a 12% higher risk of all-cause mortality, a 55% higher risk of cardiovascular mortality, and a nearly two-fold increase in stroke risk compared to men (10). Interestingly, the incidence of AF differs between men and women not only within the general population but also among individuals with MetS (11). MetS (12–14) is a constellation of metabolic conditions, such as abdominal overweight/obesity, dysglycemia, hypertension, and atherogenic dyslipidemia, which together synergistically increase the risk of developing cardiovascular diseases and AF. The increased global trends in MetS prevalence parallel the increased prevalence of AF over time (15). MetS is diagnosed when three or more of the following metabolic abnormalities are met according to the guidelines of The National Cholesterol Education Program Adult Treatment Panel III (NCEP-ATP III) (12, 13): abdominal obesity (waist circumference >102 cm for men and >88 cm for women), elevated fasting glucose (≥100 mg/dL) or a diagnosis of diabetes, increased blood pressure (≥130/85 mmHg or use of antihypertensive medications), high plasma triglycerides (≥150 mg/dL), and low high-density lipoprotein (HDL) cholesterol (<40 mg/dL in men, <50 mg/dL in women or lipid-lowering treatment).

### Atrial fibrillation in metabolic syndrome

Epidemiological studies conducted in both Asian (16–19) and Western populations (20–22) showed that individuals with MetS are more likely to have AF than those without MetS. Moreover, in the Atherosclerosis Risk in Communities Study, MetS conferred a similar risk of incident AF also in Black and White individuals (20).

In a meta-analysis of six observational studies (11), Zeng et al. reported that MetS was associated with a ∼1.6-fold increased risk of developing AF, and among MetS components, increased blood pressure (HR 1.56; 95% CI 1.46-1.66) was the predominant risk factor, followed by abdominal obesity (HR 1.37; 95% CI 1.36–1.38), elevated blood glucose (HR 1.18; 95% CI 1.15–1.21) and low HDL cholesterol levels (HR 1.18; 95% CI 1.06–1.32).

Watanabe et al. (16) studied 28,449 middle-aged subjects without baseline AF. In this study, the authors examined the role of MetS classified using either NCEP-ATP III or American Heart Association/National Heart, Lung, and Blood Institute (AHA/NHLBI) definitions on the risk of new-onset AF. The MetS was present in 13% and 16% of these subjects using either the NCEP-ATP III or AHA/NHLBI definitions, respectively. During a mean follow-up of 4.5 years, new-onset AF developed in 265 subjects (105 women and 160 men), with an incidence rate of 4.1 and 1.3 per 1,000 person-years in men and women, respectively. Notably, in multivariable Cox proportional-hazard ratio models adjusted for age and sex, the presence of MetS, as defined by either NCEP-ATP III or AHA/NHLBI definitions, significantly increased the risk of new-onset AF by nearly 1.9-fold and 1.6-fold, respectively, compared to subjects without MetS. Furthermore, the hazard ratios for new-onset AF increased sharply across the increasing number of MetS components, and the trend was similar for both MetS definitions used. When the authors examined the contribution of each MetS component to AF development, they found that all of the MetS components, except for increased plasma triglycerides, were significantly correlated with an increased risk of new-onset AF. In particular, obesity (HR 1.64; 95% CI 1.26–2.15) and increased blood pressure (HR 1.69; 95% CI 1.26–2.27) contributed the most to the increased risk of new-onset AF. Also, low high-density lipoprotein cholesterol (HR 1.52; 95% CI 1.09–2.14) and dysglycemia (HR 1.44; 95% CI 1.09–1.90) showed an increased risk of incident AF (16).

In a retrospective cohort of about 22.9 million South Korean middle-aged individuals (mean age 47.5 years, 50.7% men) whose data were obtained from the Korean National Health Insurance Service between 2008 and 2013, Choe et al. (17) found that 225,529 (0.98%) individuals developed incident AF during a mean follow-up of 5.4 years, with an overall AF incidence rate of 1.84 per 1,000 person-years. MetS was diagnosed in 27.4% of these individuals at the time of the baseline evaluation based on the NCEP-ATP III definition, whereas 26.4% presented without any of the five MetS components. After adjusting for age, sex, physical activity, alcohol consumption, and smoking status, the hazard ratio for incident AF among individuals with MetS was 1.38 (95% CI 1.36–1.39) compared to those without MetS. Each MetS component showed a significant, independent association with the risk of incident AF. In particular, increased blood pressure was the most strong predictor of AF incidence (HR 1.51, 95% CI 1.50–1.52), followed by larger waist circumference (1.37, 95% CI 1.36-1.38), low HDL cholesterol (HR 1.24, 95% CI 1.23–1.25), dysglycemia (HR 1.19, 95% CI 1.18–1.20), and increased plasma triglycerides (HR 1.12, 95% CI 1.11–1.13). The authors also reported a stepwise increase in AF risk based on the number of MetS components, and subjects presenting all five components had a nearly 2.2-fold higher risk of incident AF compared to those presenting no MetS components.

In a retrospective cohort study using data from 21,981 male individuals with a mean age of 46 years, who participated in the comprehensive health screening program at Korean Ulsan University, Kim and coworkers (18) confirmed that the presence of MetS at baseline was associated with an increased risk of AF. In Cox proportional hazard regression models adjusted for age, smoking status, regular exercise, drinking status, and CKD, the HR for AF in subjects with MetS was 1.57 (95% CI 1.04–2.38). Among the MetS components, abdominal obesity (adjusted HR 1.62, 95% CI 1.13–2.33, *P* < 0.01) was the only statistically significant risk factor after multivariable adjustment (18).

### Sex differences in AF incidence in patients with metabolic syndrome

Table 1 summarized the epidemiological evidence of sex differences in AF incidence in metabolic syndrome.

**Table 1:** Epidemiological evidence of sex differences in AF incidence in metabolic syndrome.

Study	Population/design	Main findings
Watanabe et al. (16)	28,449 middle-aged subjects without baseline AF; mean follow-up 4.5 years; 265 incident AF cases.	MetS increased new-onset AF risk by nearly 1.9-fold using NCEP-ATP III and 1.6-fold using AHA/NHLBI definitions. Men had higher AF incidence than women: 4.1 vs 1.3 per 1,000 person-years. Obesity and blood pressure contributed most; triglycerides were not significant.
Kim et al. (18)	Retrospective cohort of 21,981 Korean men; mean age 46 years.	MetS HR 1.57 for AF. Abdominal obesity was the only component that remained statistically significant after multivariable adjustment (HR 1.62). This was a male-only cohort.
Kwon et al. (19)	Nationwide South Korean cohort of about 7.8 million adults aged 30–69 years without baseline AF; mean follow-up 7.3 years; 20,708 incident AF cases.	Pre-MetS conferred a 1.4-fold higher AF risk, while MetS conferred a 1.7-fold higher risk. Men had higher AF incidence across all age decades; women showed a sharp increase after age 60. Male sex and older age were the strongest predictors.
Nyström et al. (21)	Swedish population-based cohort; 4,021 middle-aged subjects; mean follow-up 13.6 years; 285 incident AF cases.	AF risk increased progressively from overweight with MetS (HR 1.67) to obese without MetS (HR 1.75) and obese with MetS (HR 1.92), compared with normal-weight subjects without MetS. AF cases were more often men.
Vyssoulis et al. (22)	15,075 non-diabetic patients with essential hypertension; five MetS definitions used.	MetS was associated with AF independently of the definition used, and AF prevalence increased with increasing number of MetS components. No association between sex and AF was observed in this hypertensive population.
Magnussen et al. (23)	BiomarCaRE pooled analysis; 79,793 individuals without AF; median follow-up 12.6 years.	New AF occurred in 4.4% of women and 6.4% of men. Incidence increased after age 50 in men and after age 60 in women, becoming similar at older ages. BMI association was stronger in men (HR 1.31 per SD) than women (HR 1.18 per SD).
Frost et al. (24)	Danish Diet, Cancer, and Health Study; 47,589 middle-aged individuals without pre-existing cardiovascular disease; mean follow-up 5.7 years; 553 AF cases.	AF HR was 1.75 in men and 1.39 in women. Obesity HR was 2.35 in men and 1.99 in women, suggesting a sex-specific interaction between BMI and incident AF.
Knuiman et al. (25)	Busselton cohort; 4,267 adults in Western Australia; 15 years of follow-up; 343 incident AF cases.	Older age, male sex, taller height, hypertension treatment and higher BMI were major AF risk factors. The effect of BMI on AF risk was stronger in men than in women.

AF, atrial fibrillation; AHA/NHLBI, American Heart Association/National Heart, Lung, and Blood Institute; BMI, body mass index; HR, hazard ratio; MetS, metabolic syndrome; NCEP-ATP III, National Cholesterol Education Program Adult Treatment Panel III; SD, standard deviation.

Kwon et al. (19) reported data obtained retrospectively from the Korean National Health Insurance Service from 2009 to 2016, evaluating about 7.8 million men and women (between 30 and 69 years of age) without baseline AF and determining the impact of MetS on their risk of developing new-onset AF. Participants were subdivided into three subgroups depending on their number of MetS components at baseline: 0 (normal), 1–2 (pre-MetS), or 3–5 (MetS) according to the NCEP-ATP III definition. During a mean follow-up of 7.3 years, 20,708 subjects (0.26%) developed incident AF. After multivariable adjustment, the risk of AF was significantly correlated with MetS. Pre-MetS had a 1.4-fold increased risk of incident AF, and MetS had a 1.7-fold increased risk. Notably, there were higher incidence rates of AF in males compared to females across all age decades, with a sharp increase in the decades over 60 in women (19). In this study, male sex and older age were the strongest predictors for AF incidence (19). Among the individual components of MetS, abdominal obesity, increased blood pressure, and elevated fasting glucose were independently associated with an increased risk of AF in multivariable age and sex-adjusted regression models (19).

Nyström et al. (21) followed up 4,021 middle-aged subjects (mean age 60 years, 47,9% men) in a population-based cardiovascular study in Sweden. MetS was present in 1,108 participants (27.6%), and 795 (19.8%) were obese. During a mean follow-up of 13.6 years, 285 subjects developed incident AF. Compared to normal weight subjects without MetS, AF risk increased progressively in overweight subjects with MetS (HR 1.67; 95% CI 1.16–2.41), obese subjects without MetS (HR 1.75; 95% CI 1.11–2.74), and obese subjects with MetS (HR 1.92; 95% CI 1.34–2.74). Participants who developed AF were more likely to be men, and to have a higher prevalence of MetS, and higher alcohol intake. Increased blood pressure was the most common MetS component associated with AF risk, while increased triglycerides were the least common (21).

Vyssoulis et al. (22) studied 15,075 consecutive, non-diabetic patients with essential hypertension (age range: 40–95 years, 51% males). MetS was diagnosed by using ﬁve different deﬁnitions. MetS prevalence varied from 31.7% to 47.8% according to each deﬁnition used. In multiple logistic regression analysis, MetS was associated with the presence of AF, independently of the MetS deﬁnition used. Also in this study, the prevalence of AF increased progressively with the increasing number of MetS components, but no association was observed between sex and AF in this population.

Magnussen et al. (23) in a pooled analysis on 79,793 individuals without AF (median age 49.6 years, 51.7% women) from four community-based European studies of the BiomarCaRE consortium, examined the incidence of AF and its attributable risk by sex. During a median follow-up time of 12.6 years, these authors observed 4.4% new cases of AF in women and 6.4% in men. The cumulative incidence rates increased markedly after the age of 50 years in men and after 60 years in women, but became similar at older ages. Multivariable-adjusted regression models revealed sex differences for the association between body mass index and increased AF risk both in women (HR per each standard deviation increase 1.18, 95% CI 1.12 -1.23) and in men (HR 1.31, 95% CI 1.25–1.38). The risk of AF for all risk factors combined was 41.9% in women and 46% in men, but interestingly, about 20% of this risk was attributable to increased BMI.

In a population-based cohort study of 47,589 middle-aged individuals without preexisting cardiovascular disease belonging to the Danish Diet, Cancer, and Health Study, the authors identified 553 cases of AF (372 men and 181 women) developed during a mean follow-up of 5.7 years (24). The HR for AF was 1.75 (95% CI 1.35–2.27) in men and 1.39 (95% CI 0.99–1.94) in women. The adjusted HR by obesity was 2.35 (95% CI 1.70–3.25) in men and 1.99 (95% CI 1.31–3.02) in women, suggesting a sex-specific interaction of BMI with incident AF (24).

Similarly, in a cohort study of 4,267 adults in Busselton (25), Western Australia, who were followed for 15 years for incident AF, a total of 343 (8%) experienced new-onset AF during follow-up. Cox regression analysis confirmed older age, male sex, taller height, being on hypertension treatment, and higher BMI as the major common risk factors, and the effect of BMI on AF risk was stronger in men than in women.

Apart from the study by Kim et al. (18), which was restricted to male subjects, and the study by Vyssoulis et al. (22), which did not find any significant differences between sexes, all other studies observed a differential impact of sex on the risk of incident AF in both the general population and in in individuals with MetS.

### Putative mechanisms for the sex differences in AF incidence in metabolic syndrome

It is unclear what could cause the observed sex-related differences in AF risk, but it is reasonable to hypothesize that biological, social, and behavioural differences could be the basis of this difference in AF incidence between men and women. The contribution of the individual components of the MetS differs between men and women (1, 26), and this may also impact the different incidence and prognosis of AF in the two sexes (27). Biological differences primarily revolve around hormones, such as estrogens and testosterone, which shape fat distribution, lipid metabolism, and insulin sensitivity, creating distinct MetS risk patterns for men and women that may promote long-term structural and electrical atrial changes predisposing to AF development (Table 2) (28). Estrogens can stimulate the storage of subcutaneous fat rather than visceral fat. Women, especially during their reproductive years, tend to store fat subcutaneously, typically around the hips and thighs, a pattern often referred to as peripheral (“gynoid”) fat distribution (29). Subcutaneous fat is less harmful, less metabolically active, and less likely to contribute to insulin resistance and low-grade inflammation (30, 31). This peripheral fat distribution explains why premenopausal women have lower rates of cardiovascular diseases and particularly of AF incidence compared to men of the same age (1). Moreover, estrogens help maintain more favourable lipid profiles (32) by increasing HDL cholesterol and lowering LDL cholesterol and triglyceride levels, thus reducing the risk of incident AF. Additionally, estrogens can improve insulin sensitivity (33) and reduce the risk of new-onset type 2 diabetes, another important clinical risk factor for AF. However, after menopause, estrogen levels decline and often result in a shift in body fat distribution, with increased visceral fat accumulation (34), along with worsened lipid profiles (34) and increased insulin resistance, leading to a markedly increased risk of developing MetS (35, 36), thus paralleling the increasing AF incidence with aging. Interestingly, the incidence of AF is also increased in individuals with metabolic dysfunction-associated steatotic liver disease (MASLD) (37), a condition closely associated with MetS (38), which is highly prevalent in men compared to premenopausal women and rises after menopause, thus further supporting the protective effects of estrogens (39), and further justifying the sex differences in incident AF at younger ages. In postmenopausal women, the decrease in serum estrogen levels unbalances the hormonal sex steroids equilibrium towards testosterone, increasing visceral fat accumulation, which in turn favours the steep rise in AF incidence in older women (40).

**Table 2:** Putative sex-specific mechanisms.

Mechanistic domain	Sex-specific pattern	Implication for AF risk
Sex hormones and fat distribution	Estrogens favour subcutaneous peripheral fat accumulation in premenopausal women. Men preferentially accumulate central visceral fat.	Earlier visceral adiposity in men may promote earlier metabolic dysfunction, inflammation and AF susceptibility.
Menopause-related metabolic transition	After menopause, declining estrogen is associated with increased central adiposity, worsened lipid profile and greater insulin resistance.	The AF incidence gap narrows with age because older women show a delayed but steep increase in AF risk.
Lipid metabolism and insulin sensitivity	Estrogens are described as favouring higher HDL cholesterol, lower LDL cholesterol and triglycerides, and better insulin sensitivity.	This may contribute to lower AF incidence in younger women, while deterioration after menopause increases MetS and AF risk.
Testosterone and ageing in men	Testosterone may preserve muscle mass and insulin sensitivity, but ageing-related testosterone decline is linked to lower muscle mass, insulin resistance, visceral adiposity and inflammation.	The relationship is age-dependent: lower testosterone in older men is associated with higher AF risk, whereas central fat accumulation remains a major male risk pathway.
MASLD and liver-metabolic risk	MASLD is closely associated with MetS and AF. It is more prevalent in men and rises in women after menopause.	This supports a broader sex-specific metabolic-risk model beyond isolated MetS components.
Autonomic regulation	Greater sympathetic-mediated responses in men and parasympathetic-mediated responses in women. Men with MetS may have more severe autonomic dysfunction.	Greater sympathetic activation linked to abdominal adiposity may further increase AF vulnerability in men with MetS.

AF, atrial fibrillation; BMI, body mass index; HDL, high-density lipoprotein; HR, hazard ratio; LDL, low-density lipoprotein; MASLD, metabolic dysfunction-associated steatotic liver disease; MetS, metabolic syndrome.

In men, testosterone may play a protective role in metabolic health, preserving muscle mass that is associated with greater insulin sensitivity, as muscle tissue helps to reduce glucose from the blood (41). However, testosterone induces more central (“android”) body fat distribution (42), where fat is stored predominantly around the abdomen and internal organs, known as visceral fat. Visceral adipose tissue is more metabolically harmful than subcutaneous fat because it releases several pro-inflammatory cytokines and free fatty acids, which worsen insulin resistance and promote atherogenic dyslipidemia, thereby creating an earlier metabolic derangement in men than in women, which may at least partially explain the development of cardiovascular disease and particularly AF at a younger age in men (1). As men age, plasma testosterone levels naturally decline, leading to decreased muscle mass, insulin resistance, increased visceral adiposity, low-grade inflammation, and altered expression of adiposity biomarkers (42–44). In a cohort of men aged ≥80 years from the Framingham Heart Study, Magnani et al. (44) reported that a 1-SD decrease in plasma testosterone levels was associated with an HR of 3.53 (95% CI 1.96–6.37) for AF risk that was not evident at younger ages. Similarly, Lai et al. observed that lower circulating testosterone levels were associated with a greater AF prevalence in men (45). Baek et al. (46) showed that the age-adjusted HR for new-onset AF for abdominal obesity was 1.44 (95% CI 1.32–1.58) for men and 1.23 (95% CI 1.11–1.37) for women, respectively, thus further suggesting that body fat distribution exposes males and females to different AF risks. Similarly, in the study by Wang and coworkers, the age-adjusted incidence rates for AF increased more in men than in women across increasing BMI categories (47). Also, sex differences in the autonomic sympathetic drive could have an impact on AF incidence in normal (48) and MetS subjects (49, 50). Men with MetS may experience more severe autonomic dysfunction, characterized by increased sympathetic nervous system activity, and body fat distribution plays an active role in determining this difference (51). In obese men and women, plasma adrenaline levels also increased more in men than in women (52). In a study of healthy older men and women (53), noradrenalin spillover was found to be higher in men than in women and was positively related to waist circumference, suggesting that body fat distribution is a key determinant of the observed sex-related differences in sympathetic activity. As a generalisation, there is a preponderance of sympathetic-mediated responses in men and of parasympathetic-mediated responses in women that may increase the long-term risk of incident AF in males compared to females, thus impacting the AF incidence differently (54, 55).

In conclusion, MetS and each component of MetS taken singularly may contribute to the development of AF by promoting long-term structural and electrical remodelling of the heart. While both men and women with MetS are at increased risk for new-onset AF, various studies indicate that the magnitude and nature of this risk differ by sex. However, it remains unclear what could cause the observed differences between sexes, and biological or psychosocial differences are called into question.
